# The genome sequence of an ichneumonid wasp, *Amblyteles armatorius* (Forster, 1771)

**DOI:** 10.12688/wellcomeopenres.18920.1

**Published:** 2023-02-06

**Authors:** Olga Sivell, Gavin R. Broad, Natural History Museum Genome Acquisition Lab, Darwin Tree of Life Barcoding collective, Wellcome Sanger Institute Tree of Life programme, Wellcome Sanger Institute Scientific Operations: DNA Pipelines collective, Tree of Life Core Informatics collective, Darwin Tree of Life Consortium

**Affiliations:** 1 Natural History Museum, London, UK

**Keywords:** Amblyteles armatorius, ichneumonid wasp, genome sequence, chromosomal, Hymenoptera

## Abstract

We present a genome assembly from an individual male *Amblyteles armatorius* (an ichneumonid wasp; Arthropoda; Insecta; Hymenoptera; Ichneumonidae). The genome sequence is 216 megabases in span. Most of the assembly is scaffolded into 12 chromosomal pseudomolecules. The mitochondrial genome has also been assembled and is 16.6 kilobases in length.

## Species taxonomy

Eukaryota; Metazoa; Ecdysozoa; Arthropoda; Hexapoda; Insecta; Pterygota; Neoptera; Endopterygota; Hymenoptera; Apocrita; Parasitoida; Ichneumonoidea; Ichneumonidae; Ichneumoninae; *Amblyteles*; *Amblyteles armatorius* (Forster, 1771) (NCBI:txid231887).

## Background

*Amblyteles armatorius* (Forster, 1771) is a large (12–16 mm) and slender species of wasp from the family Ichneumonidae. It is the only species in its genus found in Britain ( Broad, 2016). It can be distinguished by the details of the yellow stripes on the black metasoma (abdomen beyond the first segment) and propodeum (first abdominal segment, fused to the thorax) with a pair of lateral spines. As with many species of the subfamily Ichneumoninae the sexes are strongly dimorphic, but each is distinctive. The female has an amblypygous ovipositor (short, almost concealed by the hypopygium) adapted to ovipositing in lepidopteran larvae. The larvae of *A. armatorius* are internal parasitoids of *Xestia* and *Noctua* species (Lepidoptera, Noctuidae) ( Broad *et al.*, 2018), emerging from the pupae.

*Amblyteles armatorius* is very common, with a wide but scattered distribution across Britain and Ireland. The adults can be found flying from May to July and October to November ( Prehn & Raper, 2016), with only females on the wing latterly. They can often be seen flying low among the vegetation. Adult wasps feed on sugar-rich sources such as nectar and honeydew; they are particularly common on umbellifers ( Broad *et al.*, 2018; Leius, 1960; Martin, 1986).

Hinz (1985) described the biology of this species, by breeding *A. armatorius* in captivity using caterpillars of its principal host, the Large Yellow Underwing moth, *Noctua pronuba* (Linnaeus, 1758). A single male and a female of *A. armatorius* hatched from moth puparia on 31 May and 14 June respectively and copulated on 15 June. The female was fed water and honey. It ceased feeding on 01 August. Over a month later (16 September) it was removed from the jar and offered young larvae of *N. pronuba*. It oviposited in three of them, after which the female wasp was euthanised and dissected. It contained 10 to 12 eggs on each side, with 1 to 2 mature eggs ( Hinz, 1985).

The caterpillars are parasitised in the autumn. The ichneumon larva feeds inside the caterpillar, over-wintering inside the host’s pupa. The adults emerge in late spring and copulate soon after ( Hinz, 1985). Males are relatively short-lived. Fertilised females have been found aestivating, as do *Noctua pronuba*, and hibernate, which is more usual for European Ichneumoninae. Favoured diapause sites are caves and buildings in Belgium and Netherlands, with one case of an aggregation of twenty individuals reported in September from a church crypt ( Verheyde & Quicke, 2022). A large group of females was also observed at altitude on Vogelberg mountain (Switzerland) running excitedly on a tall log stack, presumably in search of aestivation sites ( Hinz, 1985). *Amblyteles armatorius* has been reported to be infected by the fungus *Ophiocordyceps ditmarii* in Europe ( Mornand *et al.*, 2012).

The high-quality genome of *A. armatorius* was sequenced as part of the Darwin Tree of Life Project, a collaborative effort to sequence all named eukaryotic species in the Atlantic Archipelago of Britain and Ireland. Here we present a chromosomally complete genome sequence for *A. armatorius*, based on one male specimen from Luton.

## Genome sequence report

**Figure 1.**
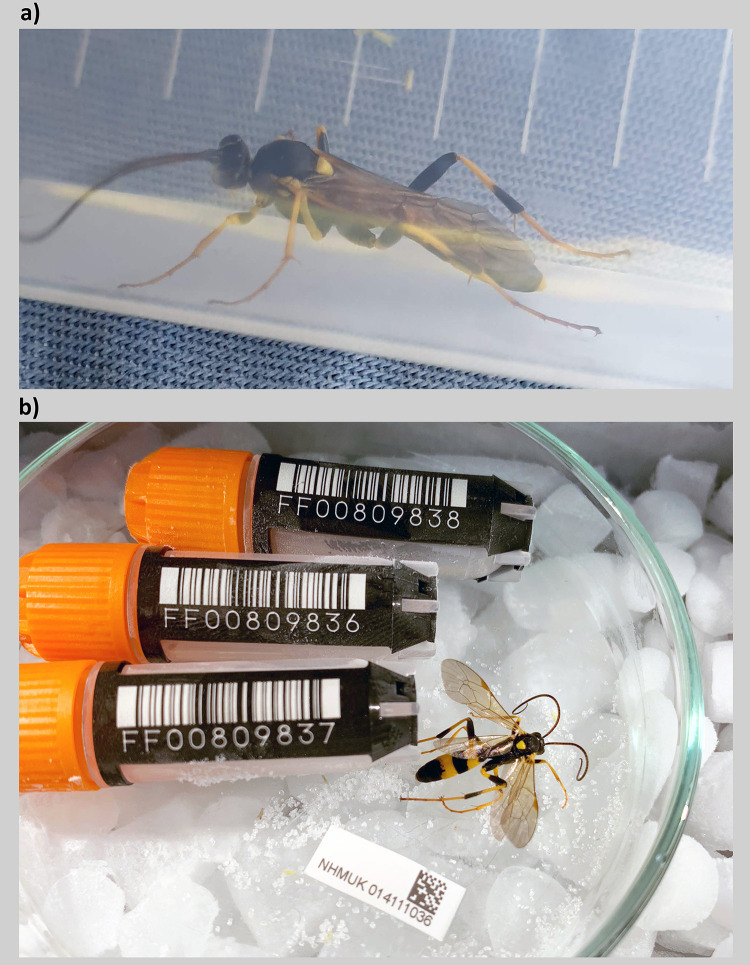
*Amblyteles armatorius* (Forster, 1771): **a**) A live specimen (NHMUK014111036) placed in a plastic tube upon collection. **b**) Image of the specimen taken during preservation and processing.

The genome was sequenced from one male *A. armatorius* ( Figure 1) collected from Luton, UK (51.88, –0.37). A total of 66-fold coverage in Pacific Biosciences single-molecule HiFi long reads was generated. Primary assembly contigs were scaffolded with chromosome conformation Hi-C data. Manual assembly curation corrected 12 missing joins or mis-joins, reducing the scaffold number by 9.09%.

**Figure 2.**
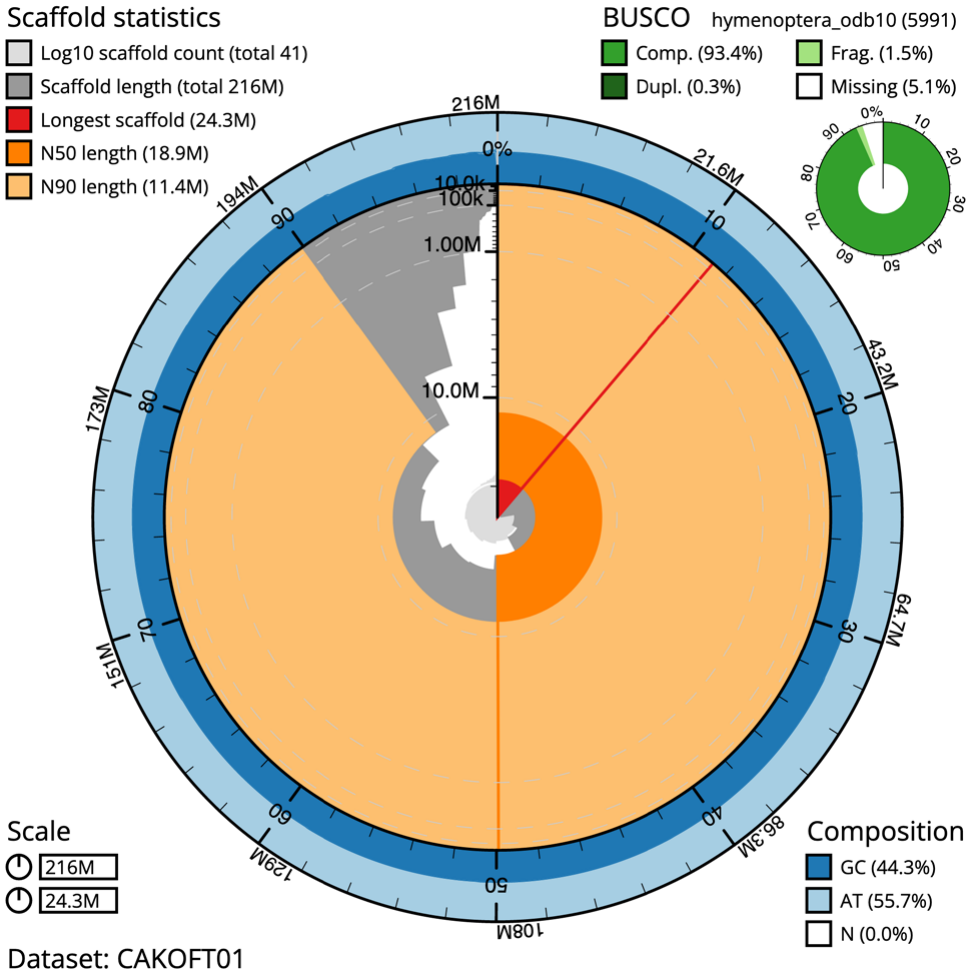
Genome assembly of *Amblyteles armatorius*, iyAmbArma2.1: metrics. The BlobToolKit Snailplot shows N50 metrics and BUSCO gene completeness. The main plot is divided into 1,000 size-ordered bins around the circumference with each bin representing 0.1% of the 215,787,317 bp assembly. The distribution of scaffold lengths is shown in dark grey with the plot radius scaled to the longest scaffold present in the assembly (24,262,552 bp, shown in red). Orange and pale-orange arcs show the N50 and N90 scaffold lengths (18,898,530 and 11,422,841 bp), respectively. The pale grey spiral shows the cumulative scaffold count on a log scale with white scale lines showing successive orders of magnitude. The blue and pale-blue area around the outside of the plot shows the distribution of GC, AT and N percentages in the same bins as the inner plot. A summary of complete, fragmented, duplicated and missing BUSCO genes in the hymenoptera_odb10 set is shown in the top right. An interactive version of this figure is available at https://blobtoolkit.genomehubs.org/view/iyAmbArma2.1/dataset/CAKOFT01/snail.

The final assembly has a total length of 215.8 Mb in 40 sequence scaffolds with a scaffold N50 of 18.9 Mb ( Table 1). Most (95.39%) of the assembly sequence was assigned to 12 chromosomal-level scaffolds, which were all autosomes. Chromosome-scale scaffolds confirmed by the Hi-C data are named in order of size ( Figure 2– Figure 5; Table 2). The assembly has a BUSCO v5.3.2 ( Manni *et al*., 2021) completeness of 93.4% (single 93.2%, duplicated 0.3%) using the hymenoptera_odb10reference set.

**Table 1.** Genome data for *Amblyteles armatorius*, iyAmbArma2.1.

Project accession data		
Assembly identifier	iyAmbArma2.1	
Species	*Amblyteles armatorius*	
Specimen	iyAmbArma2	
NCBI taxonomy ID	231887	
BioProject	PRJEB50791	
BioSample ID	SAMEA7520946	
Isolate information	male: iyAmbArma2 (PacBio, 10X); male: iyAmbArma4 (Hi-C and RNA-Seq)	
Assembly metrics *		*Benchmark*
Consensus quality (QV)	65.3	*≥ 50*
*k*-mer completeness	100%	*≥ 95%*
BUSCO **	C:93.4%[S:93.2%,D:0.3%], F:1.5%,M:5.1%,n:5,991	*C ≥ 95%*
Percentage of assembly mapped to chromosomes	95.39%	*≥ 95%*
Sex chromosomes	N/A	*localised homologous pairs*
Organelles	Mitochondrial genome	*complete single alleles*
Raw data accessions		
PacificBiosciences SEQUEL II	ERR8575397	
Hi-C Illumina	ERR8571698	
PolyA RNA-Seq Illumina	ERR8571697	
Genome assembly		
Assembly accession	GCA_933228735.2	
Span (Mb)	215.8	
Number of contigs	93	
Contig N50 length (Mb)	4.9	
Number of scaffolds	40	
Scaffold N50 length (Mb)	18.9	
Longest scaffold (Mb)	24.3	

* Assembly metric benchmarks are adapted from column VGP-2020 of “ Table 1: Proposed standards and metrics for defining genome assembly quality” from ( Rhie *et al.*, 2021). ** BUSCO scores based on the hymenoptera_odb10 BUSCO set using v5.3.2. C = complete [S = single copy, D = duplicated], F = fragmented, M = missing, n = number of orthologues in comparison. A full set of BUSCO scores is available at https://blobtoolkit.genomehubs.org/view/iyAmbArma2.1/dataset/CAKOFT01/busco.

**Figure 3.**
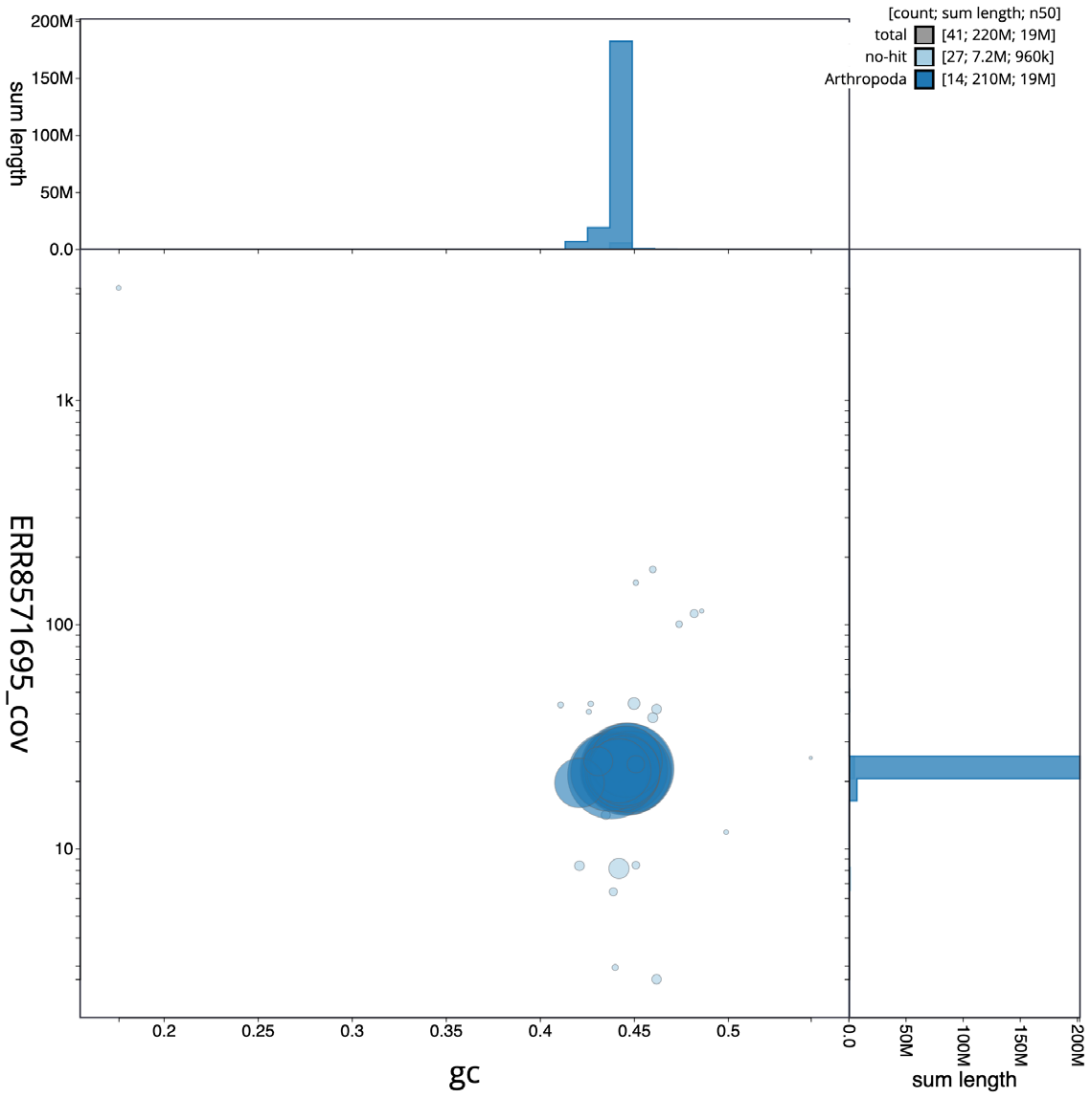
Genome assembly of *Amblyteles armatorius*, iyAmbArma2.1: GC coverage. BlobToolKit GC-coverage plot. Scaffolds are coloured by phylum. Circles are sized in proportion to scaffold length. Histograms show the distribution of scaffold length sum along each axis. An interactive version of this figure is available at https://blobtoolkit.genomehubs.org/view/iyAmbArma2.1/dataset/CAKOFT01/blob.

**Figure 4.**
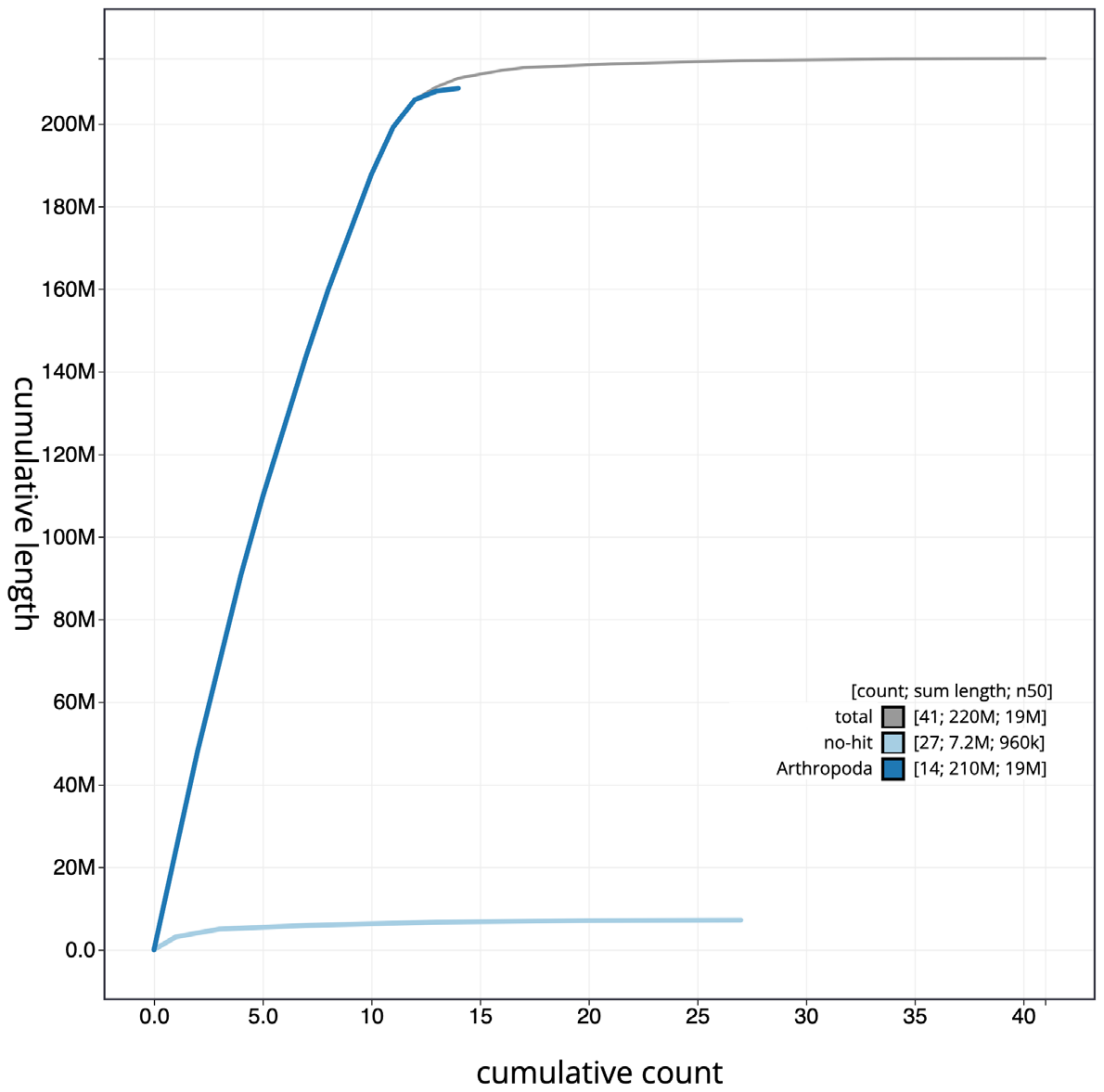
Genome assembly of *Amblyteles armatorius*, iyAmbArma2.1: cumulative sequence. BlobToolKit cumulative sequence plot. The grey line shows cumulative length for all scaffolds. Coloured lines show cumulative lengths of scaffolds assigned to each phylum using the buscogenes taxrule. An interactive version of this figure is available at https://blobtoolkit.genomehubs.org/view/iyAmbArma2.1/dataset/CAKOFT01/cumulative.

**Figure 5.**
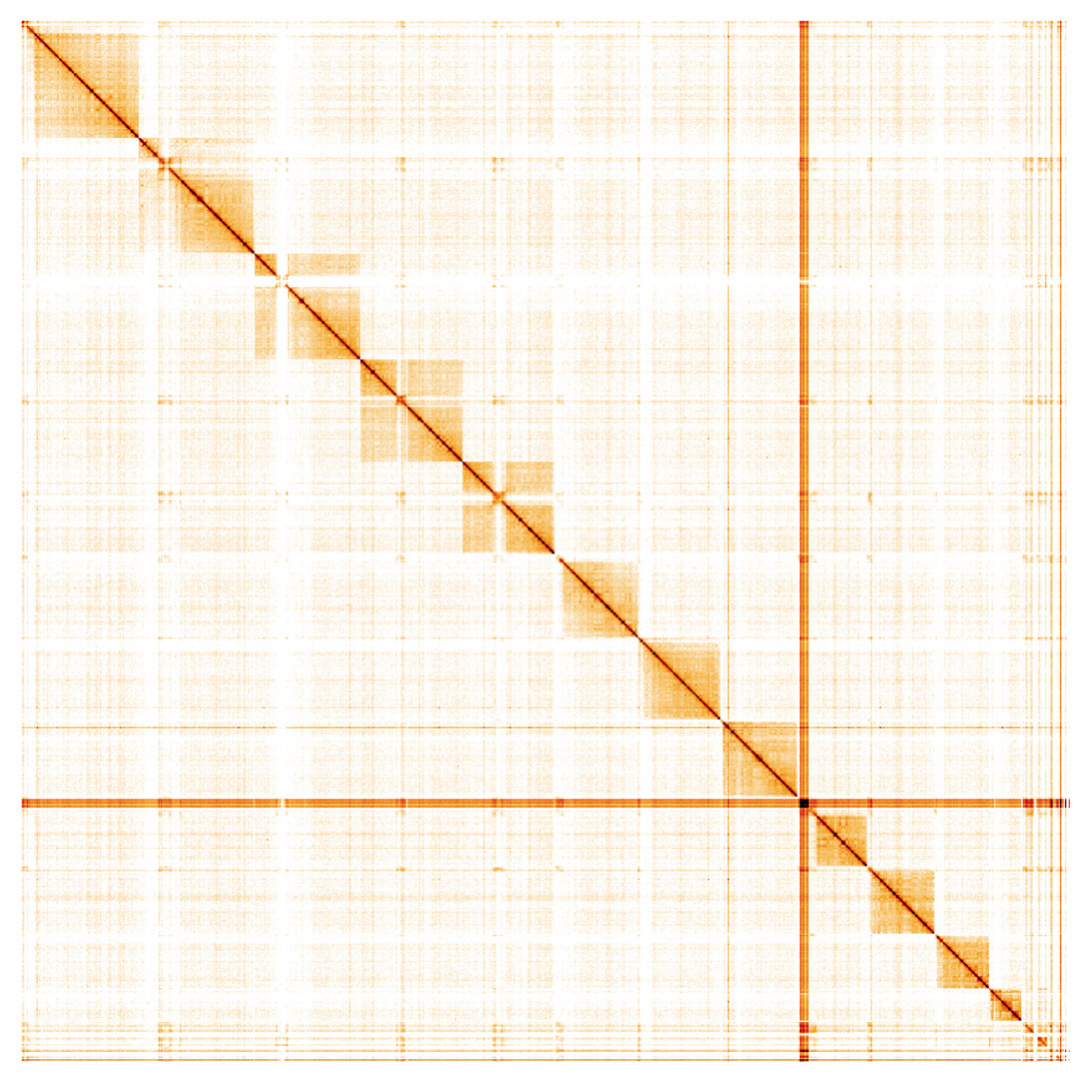
Genome assembly of *Amblyteles armatorius*, iyAmbArma2.1: Hi-C contact map. Hi-C contact map of the iyAmbArma2.1 assembly, visualised using HiGlass. Chromosomes are shown in order of size from left to right and top to bottom. An interactive version of this figure may be viewed at https://genome-note-higlass.tol.sanger.ac.uk/l/?d=ZWLIofWNSi6-MiniR9_Waw.

**Table 2.** Chromosomal pseudomolecules in the genome assembly of *Amblyteles armatorius*, iyAmbArma2.

INSDC accession	Chromosome	Size (Mb)	GC%
OW121686.1	1	24.26	44.6
OW121687.1	2	23.73	44.7
OW121688.1	3	21.79	43.8
OW121689.1	4	20.98	44.5
OW121690.1	5	18.9	44.8
OW121691.1	6	17.13	44.3
OW121692.1	7	16.99	43.7
OW121693.1	8	15.82	44.4
OW121694.1	9	14.3	44.6
OW121695.1	10	13.83	44.5
OW121696.1	11	11.42	44.2
OW121697.1	12	6.67	42.1
OW121698.1	MT	0.02	17.7

## Methods

### Sample acquisition and nucleic acid extraction

Two male *Amblyteles armatorius* specimens (iyAmbArma2; NHMUK014111036 and iyAmbArma4; NHMUK014111055) were swept using an insect net from vegetation in Wigmore Park, Percival Way, Wigmore, Luton, England (latitude 51.88, longitude –0.37). The specimen yAmbArma2; NHMUK014111036 was swept on 2 June 2020 and iyAmbArma4; NHMUK014111055 on 16 June 2020. They were collected by Olga Sivell and identified by Gavin Broad (both Natural History Museum, London) and then snap-frozen on dry ice. The tissue samples taken from the two specimens were stored in a CoolRack prior to sample preparation and genome sequencing.

DNA was extracted at the Tree of Life laboratory, Wellcome Sanger Institute (WSI). The iyAmbArma2 sample was weighed and dissected on dry ice. Thorax tissue was disrupted using a Nippi Powermasher fitted with a BioMasher pestle. High molecular weight (HMW) DNA was extracted using the Qiagen MagAttract HMW DNA extraction kit. HMW DNA was sheared into an average fragment size of 12–20 kb in a Megaruptor 3 system with speed setting 30. Sheared DNA was purified by solid-phase reversible immobilisation using AMPure PB beads with a 1.8× ratio of beads to sample to remove the shorter fragments and concentrate the DNA sample. The concentration of the sheared and purified DNA was assessed using a Nanodrop spectrophotometer and Qubit Fluorometer and Qubit dsDNA High Sensitivity Assay kit. Fragment size distribution was evaluated by running the sample on the FemtoPulse system.

RNA was extracted from thorax tissue of iyAmbArma4 in the Tree of Life Laboratory at the WSI using TRIzol, according to the manufacturer’s instructions. RNA was then eluted in 50 μl RNAse-free water and its concentration assessed using a Nanodrop spectrophotometer and Qubit Fluorometer using the Qubit RNA Broad-Range (BR) Assay kit. Analysis of the integrity of the RNA was done using Agilent RNA 6000 Pico Kit and Eukaryotic Total RNA assay.

### Sequencing

Pacific Biosciences HiFi circular consensus and 10X Genomics read cloud DNA sequencing libraries were constructed according to the manufacturers’ instructions. Poly(A) RNA-Seq libraries were constructed using the NEB Ultra II RNA Library Prep kit. DNA and RNA sequencing were performed by the Scientific Operations core at the WSI on Pacific Biosciences SEQUEL II (HiFi), Illumina HiSeq 4000 (RNA-Seq) and HiSeq X Ten (10X) instruments. Hi-C data were also generated from head tissue of iyAmbArma2 using the Arima v2 kit and sequenced on the Illumina NovaSeq 6000 instrument.

### Genome assembly

Assembly was carried out with Hifiasm ( Cheng *et al.*, 2021) and haplotypic duplication was identified and removed with purge_dups ( Guan *et al.*, 2020). The assembly was then scaffolded with Hi-C data ( Rao *et al.*, 2014) using YaHS ( Zhou *et al.*, 2022). The assembly was checked for contamination and corrected using the gEVAL system ( Chow *et al.*, 2016) as described previously ( Howe *et al.*, 2021). Manual curation was performed using gEVAL, HiGlass ( Kerpedjiev *et al.*, 2018) and Pretext ( Harry, 2022). The mitochondrial genome was assembled using MitoHiFi ( Uliano-Silva *et al.*, 2022), which performed annotation using MitoFinder ( Allio *et al.*, 2020). The genome was analysed and BUSCO scores were generated within the BlobToolKit environment ( Challis *et al.*, 2020). Table 3 contains a list of all software tool versions used, where relevant.

**Table 3.** Software tools and versions used.

Software tool	Version	Source
BlobToolKit	3.4.0	Challis *et al.*, 2020
freebayes	1.3.1-17-gaa2ace8	Garrison & Marth, 2012
gEVAL	N/A	Chow *et al.*, 2016
Hifiasm	0.16.1-r375	Cheng *et al.*, 2021
HiGlass	1.11.6	Kerpedjiev *et al.*, 2018
Long Ranger ALIGN	2.2.2	https://support.10xgenomics. com/genome-exome/ software/pipelines/ latest/advanced/other- pipelines
MitoHiFi	1	Uliano-Silva *et al.*, 2022
PretextView	0.2	Harry, 2022
purge_dups	1.2.3	Guan *et al.*, 2020
YaHS	yahs-1.1.91eebc2	Zhou *et al.*, 2022

### Ethics/compliance issues

The materials that have contributed to this genome note have been supplied by a Darwin Tree of Life Partner. The submission of materials by a Darwin Tree of Life Partner is subject to the Darwin Tree of Life Project Sampling Code of Practice. By agreeing with and signing up to the Sampling Code of Practice, the Darwin Tree of Life Partner agrees they will meet the legal and ethical requirements and standards set out within this document in respect of all samples acquired for, and supplied to, the Darwin Tree of Life Project. Each transfer of samples is further undertaken according to a Research Collaboration Agreement or Material Transfer Agreement entered into by the Darwin Tree of Life Partner, Genome Research Limited (operating as the Wellcome Sanger Institute), and in some circumstances other Darwin Tree of Life collaborators.

## Data Availability

European Nucleotide Archive: *Amblyteles armatorius*. Accession number PRJEB50791; https://identifiers.org/ena.embl/PRJEB50791. ( Wellcome Sanger Institute, 2022) The genome sequence is released openly for reuse. The *Amblyteles armatorius* genome sequencing initiative is part of the Darwin Tree of Life (DToL) project. All raw sequence data and the assembly have been deposited in INSDC databases. The genome will be annotated using available RNA-Seq data and presented through the Ensembl pipeline at the European Bioinformatics Institute. Raw data and assembly accession identifiers are reported in Table 1.

## Author information

Members of the Natural History Museum Genome Acquisition Lab are listed here: https://doi.org/10.5281/zenodo.4790043.

Members of the Darwin Tree of Life Barcoding collective are listed here: https://doi.org/10.5281/zenodo.4893703.

Members of the Wellcome Sanger Institute Tree of Life programme are listed here: https://doi.org/10.5281/zenodo.4783585.

Members of Wellcome Sanger Institute Scientific Operations: DNA Pipelines collective are listed here: https://doi.org/10.5281/zenodo.4790455.

Members of the Tree of Life Core Informatics collective are listed here: https://doi.org/10.5281/zenodo.5013541.

Members of the Darwin Tree of Life Consortium are listed here: https://doi.org/10.5281/zenodo.4783558.
