# Supplementary material for: The genome sequence of an ichneumonid wasp, Amblyteles armatorius (Forster, 1771)
Source: Wellcome Open Res. 2023 Feb 6;8:61. [Version 1] doi: 10.12688/wellcomeopenres.18920.1 (PMC10687389; doi:10.12688/wellcomeopenres.18920.1)
Supplement: Peer review history

# Reviewer report: Reviewer response for version 1

**DOI:** 10.21956/wellcomeopenres.20978.r66529

**Published:** 2023-11-29

**Authors:** Željko Tomanović

**Affiliations:** 1 University of Belgrade, Studentski trg, Belgrade, Serbia

The authors sequenced and assembled the parasitic wasp genome of *Amblyteles armatorius* (Forster, 1771). The introduction provides valuable information on various aspects of the biology of this parasitic species, including data on the host of noctuid caterpillars. I only suggest adding a sentence about the possible importance of known hosts in forestry and agriculture.

Are sufficient details of methods and materials provided to allow replication by others?

Yes

Is the rationale for creating the dataset(s) clearly described?

Yes

Are the datasets clearly presented in a useable and accessible format?

Yes

Are the protocols appropriate and is the work technically sound?

Yes

Reviewer Expertise:

Entomology

I confirm that I have read this submission and believe that I have an appropriate level of expertise to confirm that it is of an acceptable scientific standard.

# Reviewer report: Reviewer response for version 1

**DOI:** 10.21956/wellcomeopenres.20978.r63356

**Published:** 2023-08-24

**Authors:** Elisabeth A. Herniou

**Affiliations:** 1 Institut de Recherche sur la Biologie de l'Insecte (IRBI), UMR 7261, CNRS-Université de Tours, Tours, 37200, France

The authors report the chromosome scale assembly of the genome sequence of the ichneumonid wasp, *Amblyteles armatorius*, obtained using state-of-the-art methodologies. The background provides important information on the life history of the species including interactions with its hosts. As a minor comment, the authors should add one or two sentences to explain in the methods how the RNAseq data was used (automated annotation, identification of BUSCO genes?).

Are sufficient details of methods and materials provided to allow replication by others?

Partly

Is the rationale for creating the dataset(s) clearly described?

Yes

Are the datasets clearly presented in a useable and accessible format?

Yes

Are the protocols appropriate and is the work technically sound?

Yes

Reviewer Expertise:

Evolutionary biology, Insect viruses, parasitoid wasps, comparative genomics

I confirm that I have read this submission and believe that I have an appropriate level of expertise to confirm that it is of an acceptable scientific standard.
